# Advances in Molecular Dynamics Simulations for Hydrogels and Nanocomposite-Reinforced Hydrogels: Multiscale Simulation Strategies and Future Directions

**DOI:** 10.3390/gels12040288

**Published:** 2026-03-29

**Authors:** Lanlan Wang, Xiangling Gu, Yanyan Zhao, Jinju Tian, Xiaokun Ma, Mingqiong Tong

**Affiliations:** 1 School of Chemical Engineering, Jilin University of Chemical Technology, Jilin 132021, China; 2 Shandong Engineering Research Center of Novel Pharmaceutical Excipients, Sustained and Controlled Release Preparations, College of Health and Medicine, Dezhou University, Decheng District, Dezhou 253023, China

**Keywords:** hydrogels, nanocomposite−enhanced hydrogels, molecular dynamics simulation, interfacial hydration, ionic crosslinking, nonequilibrium response

## Abstract

Hydrogels and nanocomposite−enhanced hydrogels, owing to their high−water content, excellent biocompatibility, and mechanical flexibility, have demonstrated broad application prospects in tissue engineering, drug delivery, and flexible electronics. With the continuous advancement of computational power, molecular dynamics (MD) simulations have increasingly become an important tool for characterizing nanocomposite materials and hydrogel systems. This approach enables the capture of structural evolution at the atomic/molecular scale and provides mechanistic insights into deformation behaviors and interaction mechanisms under external stimuli such as mechanical force, temperature, and electric fields. This review is organized around the central framework of “structural construction–interfacial regulation−responsive behavior–dynamic evolution”, and systematically summarizes the recent progress in the application of molecular dynamics and multiscale simulation methods to hydrogels and nanocomposite hydrogels. The systems discussed mainly include synthetic polymer-based hydrogels, natural polymer−based hydrogels, peptide/protein−based hydrogels, and nanocomposite hydrogels. Particular emphasis is placed on modeling strategies and force−field selection principles for describing atomic interactions in various nanocomposite hydrogel systems. In addition, the important applications of multiscale simulation strategies in elucidating the interfacial behavior of hydrogels and the mechanisms underlying their dynamic responses under nonequilibrium conditions are also discussed. Finally, future development trends are outlined, including multiscale coupled simulations, closed−loop correction between experiments and simulations, and data−driven modeling strategies for the precise design and performance prediction of complex hydrogel systems.

## 1. Introduction

Hydrogels, as a class of soft materials constructed from three−dimensional hydrophilic polymer networks, have been widely applied in biomedical materials, tissue engineering, flexible electronics, food engineering, and environmental remediation due to their high−water content, tunable mechanical properties, and excellent biocompatibility [1,2,3]. In recent years, with the advancement of molecular design strategies and nanotechnology, hydrogel systems have continuously evolved toward enhanced functionality, structural ordering, and integrated performance [4,5]. For example, antibacterial hydrogels constructed through the self−assembly of natural antimicrobial peptides can achieve efficient inhibition of various bacteria via a “capture−kill” mechanism [6]. Protein hydrogel systems exhibiting ultrahigh stretchability and self-healing properties through regulation of the solvent environment and hydrogen−bonding network have also been systematically elucidated [7]. Janus wet−adhesive materials with asymmetric adhesion properties, along with ultralow-modulus and highly swellable PNAAA (poly(N-acryloyl alaninamide)) hydrogels, have provided new strategies for postoperative anti-adhesion and tissue repair [8,9]. These findings indicate that the macroscopic functions of hydrogels fundamentally originate from molecular-scale interactions and their hierarchical structural evolution processes.

Molecular dynamics (MD) simulation is an important tool for elucidating molecular self−assembly pathways, hydrogen-bond network reconstruction, π−π stacking behavior, and solvent-mediated regulatory mechanisms in hydrogel systems [10,11,12,13]. Numerous studies have demonstrated that MD simulations can clearly resolve the cooperative effects of aromatic stacking and hydrogen bonding on nanofiber formation in supramolecular peptide hydrogels. For example, the octapeptide GV8, derived from squid sucker ring teeth proteins, undergoes a conformational transition from a 3_10_−helix to an antiparallel β−sheet during assembly, and its mechanism of mechanical reinforcement has been supported by simulation results [14]. In co-assembled systems of L/D enantiomeric tripeptides, hydrogen bonding and π−π stacking interactions within heterodimers have been confirmed to be key factors governing supramolecular chirality [15]. The self-assembly mechanisms of Fmoc-modified peptide hydrogels, as well as their synergistic reinforcement mechanisms with nanomaterials such as graphene, have also been elucidated through simulations [16,17,18]. In addition, studies on the pH−triggered self-assembly of biphenyl tripeptides have further clarified the central role of the “FF” sequence and hydrogen−bonding interactions in the gelation process [19].

In protein-based and multicomponent systems, MD simulations also play a crucial role [20,21]. For example, in polyphenol–protein systems, the competitive hydrogen bonding between water molecules and polyphenol molecules has been shown to inhibit β−sheet formation and enhance network reconfigurability [7]. In studies of protein crystallization using hydrogel templates, MD simulations have revealed the stabilizing role of α-helical structures in protein nucleation and crystal growth [22]. The molecular interaction mechanism between puerarin and whey protein has also been elucidated through spectroscopic analysis and molecular docking studies, providing a theoretical basis for the design of functional food gels [23]. Meanwhile, nanoreinforcement strategies have provided a new dimension for enhancing hydrogel performance. By incorporating conductive polymers, carbon nanomaterials, or graphene fibers, multiscale synergistic networks can be constructed to integrate electrical conductivity, thermal transport, and mechanical reinforcement. For example, a conductive composite hydrogel based on RGD (Arg–Gly–Asp) short peptides and polyaniline has demonstrated excellent bioelectrical coupling performance in cardiac tissue engineering [16]. The PVA/CS composite hydrogel containing PPy−PDA nanoparticles exhibits excellent photothermal responsiveness and electrical conductivity [24]. Hierarchical PVA hydrogels reinforced with oxygen plasma−treated graphene fibers achieve significant improvements in thermal conductivity and mechanical performance [25]. Amino acid self-assembled hydrogels doped with oxidized carbon nanotubes or graphene oxide have achieved light−controlled drug release functionality [26]. Molecular simulations have provided critical theoretical support for elucidating the interfacial interaction modes and energy dissipation mechanisms between nanofillers and polymer chains.

In the fields of biomedicine and tissue engineering, molecular dynamics simulations have also been employed to elucidate the molecular basis of hydrogels in antibacterial activity, hemostasis, anti-adhesion, and tissue regeneration. For example, in CS/PPP (chitosan/poly (D,l-lactide)-poly (ethylene glycol)-poly (D,l-lactide)) thermosensitive hydrogels, hydrogen bond−driven long−chain structures endow the system with rapid gelation and hemostatic properties [27]. In branched starch−tannic acid hydrogels, the synergistic effects of high−density hydrogen bonding and van der Waals interactions enhance mechanical strength and self−healing capability [28]. The dynamic RS−Ag coordination interactions endow conductive hydrogels with excellent self−healing capability and multi-responsive properties [29]. High−strength protein hydrogels constructed via tripeptide-gelatin co-assembly have demonstrated promising potential in cartilage regeneration [30]. In addition, the application of carrier-free drug self−assembled hydrogels in the prevention of postoperative tumor recurrence [31], as well as the performance of naturally derived small-molecule co−assembled hydrogels in anti−inflammatory and antibacterial therapies [32], both underscore the decisive influence of molecular-level interactions on macroscopic therapeutic outcomes.

In recent years, PVA (polyvinyl alcohol)−based composite hydrogels have shown promising antibacterial properties and potential applications in promoting wound healing through the incorporation of natural bioactive components or functional nanomaterials. Chopra et al. developed a curcumin-loaded chitosan–PVA hydrogel and demonstrated that it exhibited significant antibacterial activity against both Gram-positive and Gram-negative bacteria. Molecular docking results further revealed that the curcumin components showed favorable binding energy scores and significant interactions with key residues of inflammation-related proteins, which may contribute to enhanced wound healing [33]. A double−network composite hydrogel composed of PVA, sodium alginate (SA), and tannic acid (TA) also exhibited favorable antibacterial activity. The study showed that the hydrogel without tannic acid treatment had relatively weak antibacterial effects, particularly a small inhibition zone against *Escherichia coli*. In contrast, after immersion in tannic acid, the material displayed significantly enhanced antibacterial activity against both *E. coli* and Staphylococcus aureus. The antibacterial mechanism was mainly attributed to the chelation of iron ions by the catechol groups in tannic acid and dopamine, which can restrict the supply of iron essential for bacterial growth. In addition, tannic acid can interact with peptidoglycan in the bacterial cell wall, thereby increasing cell wall permeability and disrupting its structural integrity, ultimately leading to bacterial death [34]. A physically crosslinked hydrogel composed of PVA, graphene−based materials (GBM), and aloe vera (Av) exhibited strong antibacterial activity against Staphylococcus aureus, with the PVA/GO group showing the most prominent effect and achieving a bacterial reduction rate of 99.94% [35].

Therefore, constructing a unified molecular−scale theoretical framework centered on the physical nature of confined water states, the synergistic mechanisms of interfacial charge–hydration interactions, and external field−driven nonequilibrium reconstruction pathways has become a core task in current molecular simulation research. This review addresses existing methodological bottlenecks and discusses recent advances in molecular dynamics and other multiscale simulation approaches for elucidating hydrogel network formation, interfacial structure regulation, stimulus−responsive behavior, and nonequilibrium dynamic processes. The aim is to provide theoretical guidance for the precise construction and performance optimization of complex functional hydrogel systems.

## 2. Hydrogel Bulk Networks and Mechanical Foundations Constructed by Water–Polymer–Ion Synergistic Interactions

### 2.1. Molecular States and Dynamic Characteristics of Water in Hydrogels

**Figure 1:**
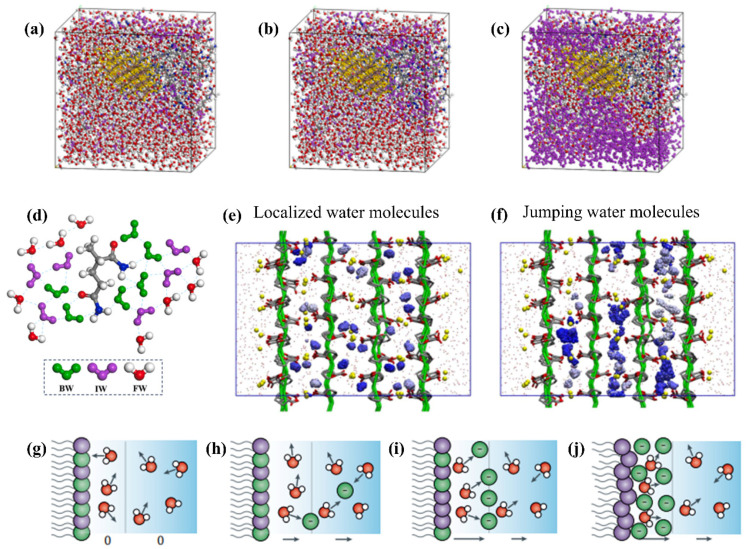
Schematic model of water molecular state distribution in hydrogels. (**a**–**c**) Bound water (BW), intermediate water (IW), and free water (FW), respectively [36]. Reprinted with permission from Elsevier of Copyright 2025. Reproduced with permission from [36]. Copyright 2025, Elsevier. (**d**) Structural illustration of the distribution of the three types of water near hydrophilic sites shown in (**a**–**c**) [36]. The blue ball represents the nitrogen atom. Reprinted with permission from Elsevier of Copyright 2025. (**e**,**f**) Two existence modes of “ultraslow water”: localized (**e**) and jumping (**f**) states [37]. Reprinted with permission from Elsevier of Copyright 2022. (**g**–**j**) Orientation and distribution of water on charged biological membranes [38]. Reprinted with permission from Springer Nature Limited of Copyright 2021.

Water in hydrogels is not a homogeneous, inert continuous medium; rather, under the influence of polymer networks, ionic environments, and interfacial interactions, it exhibits multiscale molecular organization and dynamic heterogeneity across different states. The distribution of molecular states and their dynamic behaviors directly determine the swelling behavior, stimulus responsiveness, and functional performance of hydrogels, as illustrated in Figure 1.

Ionic functional groups can systematically regulate the distribution of water molecular states by enhancing osmotic effects and water–polymer interactions [39,40,41]. Consistent results from low-field nuclear magnetic resonance (LF−NMR), ζ-potential measurements, and molecular dynamics simulations indicate that, after introducing carboxymethyl functional groups onto chitin chains, increasing the degree of substitution enhances both the mobility and binding capacity of water molecules, while significantly improving the swelling performance and pH responsiveness of the network [42]. Interfaces and external fields also serve as important approaches for regulating the behavior of water molecules. Interfacial charges can significantly rearrange the orientational distribution and hydrogen-bonding network of interfacial water, and generate strong correlation effects with specific ions, thereby revealing the limitations of classical mean-field models at the molecular scale [38]. From the perspective of electronic structure, at water–metal interfaces, the competition between water–water and water–metal interactions, together with pronounced electronic polarization effects, collectively determines the structural characteristics and dynamic behavior of interfacial water [43].

To address the longstanding debate over whether the slowing of water dynamics originates from network pore−size confinement, combined ultrafast infrared spectroscopy and MD simulations have shown that, at identical amide group concentrations, the orientational relaxation and hydrogen−bond network dynamics of water molecules in acrylamide monomer solutions, linear polymer solutions, and crosslinked hydrogel systems exhibit a high degree of consistency within experimentally accessible timescales. These results indicate that the overall slowdown of water dynamics primarily arises from local perturbations of the water hydrogen−bond network induced by amide functional groups, rather than being governed by pore size or geometric confinement effects of the hydrogel network [44]. Studies on photocatalytic functional hydrogel systems further demonstrate that bound water, intermediate water, and free water are not uniformly distributed within the gel network; instead, their distribution is regulated by nanofillers and catalytically active sites, leading to significant local enrichment and redistribution of their relative proportions [36]. In ionically crosslinked systems, fast field−cycling nuclear magnetic resonance (FFC−NMR) relaxometry combined with molecular dynamics simulations has revealed the presence of a population of “ultraslow water” strongly interacting with polymer chains in Ca^2+^− and Zn^2+^−crosslinked polygalacturonate hydrogels. The diffusion and rotational dynamics of this water population are significantly slower than those of bulk water. These findings indicate that divalent ions not only construct and regulate the coordination structure and association morphology of polysaccharide networks, but also profoundly alter the local dynamic behavior of water by enhancing polymer–water interactions [37]. The dynamic characteristics of water molecules and their regulatory factors are summarized in Table 1.

Therefore, the diffusion, orientational relaxation, and hydrogen−bond rearrangement of water molecules are not primarily governed by gel pore size or macroscopic geometric confinement, but rather dominated by the local energy landscape collaboratively shaped by functional group−water hydrogen bonding, interfacial charge distribution, and network topology. From a statistical thermodynamics perspective, a hydrogel system can be regarded as a multiminima system confined within an energy landscape; the application of external fields effectively modulates the curvature of the potential energy surface and the distribution of transition pathways, thereby altering the kinetic routes for barrier crossing. Within this theoretical framework, water states with different degrees of confinement (such as bound water and ultraslow water) can be understood as direct manifestations of changes in water–water and water–polymer interaction energies and the reorganization of orientational degrees of freedom.

### 2.2. Synergistic Regulation of Hydrogel Network Structure and Mechanical Properties by Functional Groups and Crosslinking Mechanisms

The formation and evolution of hydrogel network structures are highly dependent on the types and spatial distribution of functional groups, as well as the specific crosslinking mechanisms employed [53]. By precisely regulating interchain interactions at the molecular scale, these factors directly shape the geometric topology and energy dissipation modes of the network, thereby determining the mechanical strength, environmental stability, and biological activity of the material.

In ionically crosslinked systems, the specific coordination between functional groups and metal ions is a key factor in controlling network density and structural stability. Single−molecule atomic force microscopy experiments combined with molecular dynamics simulations have shown that the coordination of Ca^2+^ with carboxyl and sulfate groups on polysaccharide chains (infernan) not only induces local chain conformational rearrangements but also significantly enhances the elastic modulus at the microgel scale through interchain bridging [54]. Using imidazolidinyl urea as the functional backbone, a primary network can be constructed through multiple hydrogen bonds, while introducing Ni^2+^/Zn^2+^−ligand dynamic metal coordination as tunable crosslinking nodes. Molecular dynamics (MD) and density functional theory (DFT) calculations demonstrate that the strength of metal–ligand coordination interactions play a decisive role in network stability, energy dissipation behavior, and overall mechanical performance. Meanwhile, this ion-coordination network endows the material with intrinsic antibacterial activity, which is closely associated with polymer–bacterial membrane interfacial interactions and reactive oxygen species (ROS)−induced oxidative stress processes [55].

In addition, neutral functional groups (such as phenolic hydroxyl groups) can also significantly enhance the structural stability of hydrogel systems by constructing dense physically crosslinked networks. The phenolic hydroxyl groups in polyphenolic puerarin molecules form abundant hydrogen bonds with low-acyl gellan gum chains, introducing additional physical crosslinking points into the network and rendering the gel structure more compact. As a result, the elastic modulus and thermal stability of the hydrogel are markedly improved, demonstrating the mechanism by which neutral functional groups reinforce the network through enhanced hydrogen-bonding interactions [56]. Lipoic acid and kaempferol construct a dynamically crosslinked network through reversible hydrogen bonding and coordination interactions. MD simulations indicate that stable hydrogen bonds can form between the polyphenolic hydroxyl groups of kaempferol and the carboxyl groups of lipoic acid, while the aromatic ring structure exerts a conformational ordering effect on the lipoic acid chains. These findings elucidate, at the atomic scale, the intrinsic mechanism underlying the synergistic enhancement of network stability, wet tissue adhesion, and dynamic disulfide bond behavior. On this basis, further loading with typhaneoside endows the system with hemostatic, anti-inflammatory, and pro-angiogenic functions, enabling suture−free wound closure and efficient healing of diabetic infected wounds [57]. In addition, spontaneous crosslinking of the system can be achieved through dimeric hydrogen bonds formed between the surface ligands of gold nanoclusters and the acrylic acid groups of carbomer. MD simulations confirm that this hydrogen−bonding network not only maintains appropriate viscoelasticity of the hydrogel but also stabilizes the highly protonated state of the gold nanocluster ligands, thereby reducing electrostatic repulsion with bacterial cell walls and enhancing antibacterial activity. Furthermore, this dynamic noncovalent network can synergistically modulate inflammatory responses, promote macrophage polarization toward the M2 phenotype, and accelerate the healing process of infected wounds [58].

The transition from physical entanglement to covalent crosslinking is an effective strategy for achieving qualitative changes in the macroscopic response of materials. Through methacrylation modification followed by photo−crosslinking reactions, hydrogen−bonding patterns and chain conformations are reregulated, transforming glycol chitosan from a network dominated by flexible entanglements into a load-bearing structure governed by covalent crosslinks. This transformation significantly enhances material stiffness and reshapes its viscoelastic response behavior [59]. In protein hydrogel systems, Zhang et al. revealed the synergistic regulatory mechanism among the distribution of transglutaminase-induced covalent crosslinking sites, crosslinking degree, and temperature. At moderate crosslinking levels, dispersed covalent crosslinking points promote the spatial proximity of triple-helix-like structures and enhance conformational compactness. In contrast, under high crosslinking conditions, covalent bonds further form a backbone-supported structure characterized by “head−to−tail” connections, significantly suppressing molecular self-assembly at elevated temperatures and reducing the binding energy of water molecules. As a result, the gelatin hydrogel exhibits stable gelation capability and thermal irreversibility [60].

Therefore, the network structure and mechanical properties of hydrogels are not determined solely by the number of crosslinks as a single parameter, but are collectively governed by a multilevel network topology and energy landscape shaped synergistically by ionic coordination, functional group types, and their spatial distribution.

## 3. Interfacial Charge−Hydration Synergy and Biological Functions of Zwitterionic Hydrogels

Focusing on the interfacial functions of zwitterionic and strongly charged hydrogel systems, related studies have systematically revealed the central roles of interfacial ion distribution, water molecular reorientation, and polymer conformational regulation in interfacial adhesion, molecular recognition, and interfacial stability [61]. In investigations of interfacial mechanisms in strongly charged systems, Shen et al. reported that divalent anions can simultaneously form electrostatic coordination interactions with multiple lysine−NH_3_^+^ sites on adjacent lysozyme nanofibers, thereby constructing stable “ionic bridges” between fibers and driving the transformation of nanofibers from a dispersed state into a three−dimensional network structure. MD simulations confirmed at the atomic scale that this ionic bridge configuration significantly lowers the system’s free energy, serving as an important physical foundation for universal gelation behavior. Building upon this structural framework, the further introduction of Mn^2+^ enabled sensitization of the cGAS−STING signaling pathway, expanding the ionically crosslinked network into an anti−recurrence hydrogel system with immune memory functionality [62]. Similarly, chitosan and carboxymethyl chitosan, as structurally analogous cationic and anionic polyelectrolytes, can form a three−dimensional physically crosslinked network through electrostatic complexation. Embedded silver nanoparticles impart stable antibacterial activity to the hydrogel without the need for chemical reducing agents or covalent crosslinking. MD simulations further indicate that the high structural similarity between polymer chains leads to favorable interfacial compatibility, which facilitates rapid reconstruction of electrostatic interactions and hydrogen−bonding networks. This provides the molecular basis for the rapid self-healing behavior observed in such hydrogels [63].

**Figure 2:**
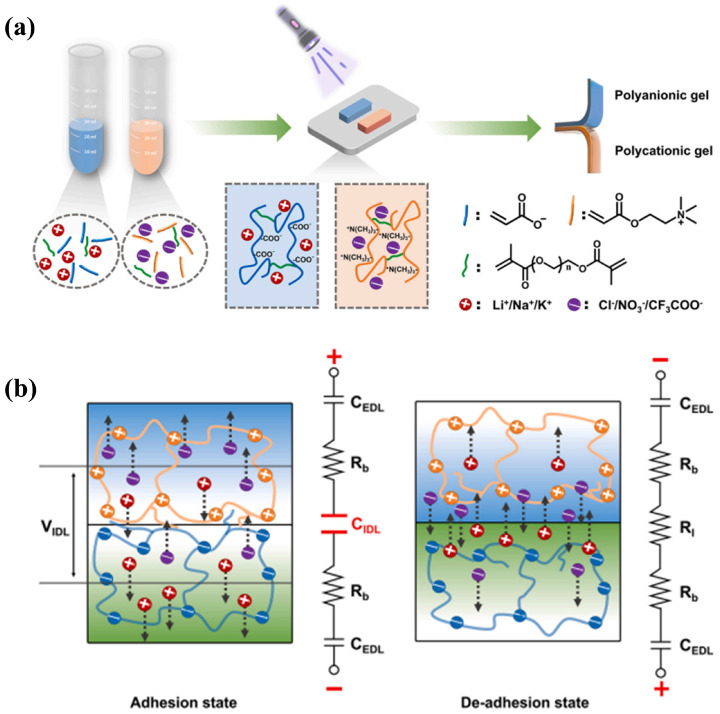
Schematic illustration of the preparation of polyanionic and polycationic hydrogels (**a**); schematic illustration of the reversible electroadhesion mechanism (**b**) [64]. The two steps in panel (**a**) are as follows: Five anionic and cationic monomers were first synthesized via an ion exchange method. Subsequently, three anionic hydrogels and three cationic hydrogels were successfully prepared through photoinitiated polymerization. Reprinted with permission from Elsevier Inc of Copyright 2025.

In terms of external field-regulated interfacial adhesion behavior, Kan et al. used a polyanion/polycation hydrogel heterogeneous interface as a model system to systematically elucidate the counterion-mediated electroadhesion mechanism (Figure 2) [64]. Under low applied voltage conditions, an ionic double layer dominated by counterion migration and rearrangement forms at the polyanion/polycation contact interface. The formation of this double layer significantly reduces interfacial ionic resistance and increases double-layer capacitance, thereby enhancing interfacial electrostatic attraction and enabling reversible, rapidly responsive electroadhesion behavior [64]. This study further distinguished the respective roles of bulk ion migration and interfacial ion rearrangement in functional performance, providing clear molecular design principles for the interfacial integration of zwitterionic hydrogels in flexible electronic devices and soft robotics.

Beyond electrically responsive adhesion, interfacial regulation in hydrogels also plays a central role in biohybrid systems, where the organization of the polymer network determines how biomacromolecules are spatially presented and functionally activated at interfaces. In star PEG−heparin hydrogels, direct analysis of the nanoscale network structure revealed that heparin retains its functionally essential rod−like conformation after incorporation into the PEG network. Consequently, it preserves its high negative charge and remains capable of effectively binding growth factors while maintaining their biological activity. Further studies showed that crosslinking density not only determines the macroscopic mechanical properties of the hydrogel, but also regulates the spatial distribution and accessibility of heparin in both bulk and interfacial regions by influencing network uniformity and defect density. These findings provide structural insight into the cell−instructive functions of biohybrid hydrogels in cell adhesion, migration, and signaling regulation [65].

**Figure 3:**
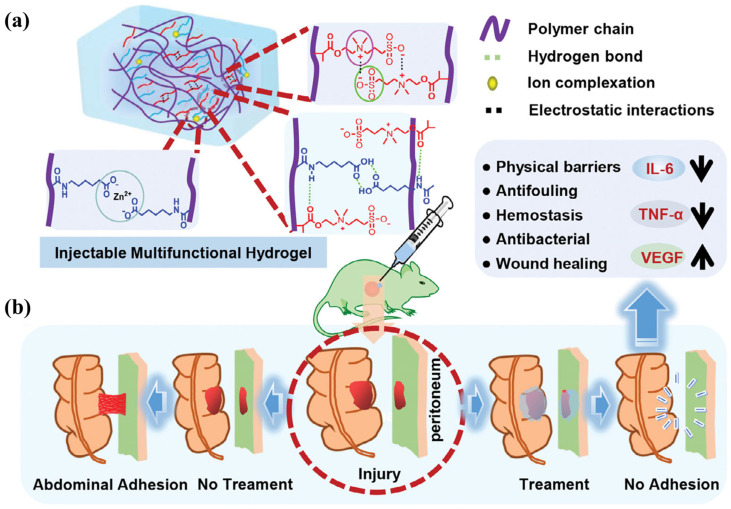
Schematic illustration of the multiple intermolecular interactions within the PSA−ZnO hydrogel (**a**). PSA−ZnO hydrogel applied in a rat cecum−abdominal wall adhesion model (**b**) [66]. The arrows in panel (**b**) indicate the multiple functional pathways of the hydrogel in vivo, leading to the final "no adhesion" effect. Reprinted with permission from Wiley−VCH GmbH of Copyright 2025.

**Figure 4:**
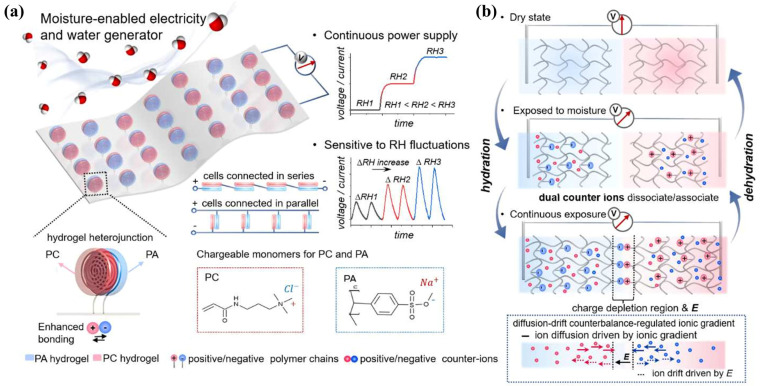
Schematic illustration of a moisture−enabled electricity and water harvesting device (MEWG) constructed from a hydrogel heterostructure. The device consists of two oppositely charged hydrogels: the positively charged PC hydrogel and the negatively charged PA hydrogel. The subpanel “Moisture-enabled electricity and water generator” in (**a**) represents the overall structure of the hydrogel heterojunction, which is formed by directly laminating a positively charged hydrogel (PC) and a negatively charged hydrogel (PA). The subpanel “Continuous power supply” in (**a**) represents the output characteristics of the hydrogel heterojunction under constant humidity, showing the variations in voltage/current over time under three different relative humidity levels. The subpanel “Sensitive to RH fluctuations” in (**a**) shows the synchronous changes in voltage/current with increases and decreases in humidity. Δ*RH* indicates the magnitude of humidity variation, and the device generates corresponding electrical signals in response to humidity fluctuations of different magnitudes (Δ*RH1*, Δ*RH2*, Δ*RH3*). The subpanel “Chargeable monomers for PC and PA” displays the sources of fixed charges in the two types of hydrogels. Schematic illustration of the moisture-driven power generation mechanism based on the hydrogel heterostructure (**b**) [69]. Reprinted with permission from Elsevier Ltd of Copyright 2022.

Among the various interfacial factors involved, hydration structure is particularly important for zwitterionic hydrogels because it directly governs both interfacial stability and bioinert behavior. Focusing on the hydration mechanism at zwitterionic interfaces, related studies have enhanced the mechanical stability of zwitterionic hydrogels by incorporating laponite nanosheets and, through the formation of a dense and stable hydration layer by SBMA segments, constructed an effective “water barrier” at the molecular scale, thereby significantly inhibiting protein adsorption and cell adhesion. The stable injectable entangled network, constructed through multiple intermolecular interactions such as hydrogen bonding and electrostatic interactions, endows the PSA−ZnO hydrogel with the ability to significantly inhibit rat peritoneal adhesions at both 7 and 14 days postoperatively (Figure 3) [66]. MD simulations further revealed that the ion–dipole interaction-dominated hydration mechanism is the fundamental origin of their antifouling and anti-adhesion properties. In vivo experiments demonstrated that this hydrogel not only serves as a physical barrier but also suppresses inflammatory responses and fibrosis progression by regulating the TGF-β/Smad signaling pathway [67]. Similarly, Chen et al. proposed an anti-dehydration hydrogel design strategy based on zwitterionic oligomers. By incorporating MPC oligomers with a molecular weight of approximately 3 kDa, the system significantly enhanced its water−binding capacity while avoiding the risk of skin penetration. Molecular simulations showed that the stable hydration layer formed between zwitterionic groups and water molecules effectively suppresses water evaporation without compromising ionic conductivity, enabling the hydrogel to maintain stable electrophysiological signal output over extended periods under low−humidity conditions [68]. Taking the cationic–anionic heterostructured hydrogel as an example, it can simultaneously harvest electrical energy and freshwater from the atmosphere (Figure 4) [69].

Taken together, these studies show that hydrogel interfacial behavior is governed not only by electrostatic interactions and network organization, but also by molecular hydration and structural reconstruction at soft matter interfaces. This broader perspective can be extended beyond solid hydrogel interfaces to complex fluid and biological interfaces. For example, in studies of complex fluid interfaces and biological interfacial stability, Shao et al. systematically compared the adsorption behaviors of protein−derived two−dimensional fibrils and three−dimensional hydrogel aggregates at oil–water interfaces. Experimental characterization combined with MD simulations demonstrated that the spatial conformational dimensionality, secondary structure composition, and the distribution and rearrangement capability of hydrophobic domains collectively determine the interfacial adsorption kinetics and interfacial reconstruction modes. Two−dimensional fibrils, owing to their higher surface hydrophobicity and greater conformational flexibility, more readily achieve rapid spreading at the interface. In contrast, three−dimensional hydrogel aggregates, once formed at the interface, are more effective in reducing interfacial tension and constructing a highly viscoelastic interfacial layer, thereby significantly enhancing interfacial rheological stability [70].

Therefore, the functional performance of zwitterionic hydrogels is not merely an extension of their bulk mechanical properties or swelling behavior, but rather highly dependent on the synergistic reconstruction of interfacial charge distribution, ion migration, and hydration structures. Accordingly, under external stimuli such as periodic electric fields, shear flow, or biological signals, the dynamic response and real-time evolution mechanisms of zwitterionic hydrogel interfacial structures will become important research directions and key focal points in this field.

## 4. Multiscale Mechanisms of Hydrogel Dynamic Responses Under External Fields and Nonequilibrium Conditions

### 4.1. Hydrogel Response Mechanisms Under External Fields and Low-Temperature Conditions

Focusing on the regulatory mechanisms of electric fields, magnetic fields, temperature, and pH on hydrogel structure and properties, related studies have systematically elucidated at the molecular scale how external fields drive significant structural reconstruction and functional responses in hydrogels by altering water molecular orientation, ion distribution, molecular conformation, and self−assembly pathways (see Table 2). As shown in Table 2, although the external fields differ, they all regulate the behavior and responsiveness of hydrogels through three common mechanisms: altering intermolecular interactions (such as hydrogen bonding, van der Waals interactions, and electrostatic interactions), modulating chain−segment mobility, and changing the network topology. The pH field acts directly on the molecular charge state and is therefore well suited for regulating the response of hydrogels to chemical environments; however, its effectiveness often depends on the design of ionizable groups within the system. The temperature field, as the most commonly used external stimulus, can influence chain mobility, hydrogen bonding, and diffusion, and can also couple with volume fraction, water content, stretching, or nanoparticle concentration, although its selectivity is sometimes less precise than that of pH. The magnetic field can dynamically regulate molecular vibrations and chain orientation in real time through an applied alternating field, making it suitable for constructing dynamically responsive systems, provided that the system possesses a certain degree of magnetic responsiveness. Hydrostatic pressure and structurally regulating external fields are more oriented toward material reinforcement, emphasizing densification, aggregation, semi-interpenetrating network formation, and enhanced chain entanglement. In contrast, multifield−coupled systems aim to simultaneously optimize mechanical properties, swelling, diffusion, and functional release. For example, triple−responsive systems involving temperature, pH, and CO_2_, as well as the synergistic optimization of permeation and drug release through the combination of temperature and nanoparticle concentration, both demonstrate that coupled external fields are better suited for constructing smart hydrogels for complex application scenarios. Molecular dynamics is the dominant approach for investigating the regulatory behavior of hydrogels, and the choice of force field exhibits clear material dependence across different systems. However, when the research focus involves larger-scale self−assembly and network formation, multiscale simulation methods such as CG-MD are more advantageous.

Molecular dynamics studies indicate that, under identical interfacial heat input conditions, the evaporation rates of hydrogels and pure water show no significant difference; moreover, the interaction energy difference between intermediate water and free water is limited, which alone cannot explain the observed enhancement in evaporation. Further simulations reveal that an alternating electric field can significantly increase the evaporation rate by inducing vibrations of water molecules and water clusters, thereby perturbing and disrupting the interfacial hydrogen-bond network, whereas a direct current electric field does not exhibit a similar effect. By disturbing the interfacial structure, hydrogels promote the formation of water clusters, which are more prone to rupture and escape from the interface under electromagnetic oscillation, manifesting the so-called “photo-molecular effect” [71]. In addition, studies on the influence of magnetic field frequency (0.01–0.05 fs^−1^) on the thermal, mechanical, and swelling behaviors of polyacrylamide (PAM) hydrogels indicate that, as the magnetic field frequency increases, the structural volume of the hydrogel decreases from 356,985 Å^3^ to 349,982 Å^3^, suggesting that polymer chain alignment becomes more ordered and the network structure more compact. Meanwhile, the ultimate strength increases slightly from 0.0325 MPa to 0.0331 MPa, while the Young’s modulus remains nearly unchanged. The heat flux and thermal conductivity increase from 1423 W/m^2^ and 0.54 W/m·K to 1472 W/m^2^ and 0.56 W/m·K, respectively. The underlying mechanism lies in the oscillating magnetic field enhancing chain segment vibrations and intermolecular interactions, thereby optimizing energy transfer pathways [72]. These results demonstrate that magnetic field frequency can serve as an effective external parameter for regulating the thermomechanical coupling properties of hydrogels, providing a theoretical basis for the design of magnetically responsive smart materials.

**Table 2:** Types of external field responses and simulation methods employed.

External Field Type	Key Function	Mechanism	Simulation Method and Force Field Type	Reference
Hydrostatic pressure field	Regulate hydrogel compactness and mechanical strength	Increased pressure alters coordination number; detachment of ionic liquids promotes cellulose bundle aggregation	MD simulation; GLYCAM06 + OPLS series + GAFF hybrid force fields	[73]
Temperature field + pH field + CO_2_	Triple-responsive hydrogel (tunable rheology)	Surfactant self-assembly structure transition; pH regulates ionization degree; CO_2_ induces reversible protonation/deprotonation	MD simulation; GROMOS 54A7 united-atom force field	[74]
pH field	Controlled self-assembly for drug-sustained-release hydrogel	Different deprotonation degrees alter molecular charge and hydrophilicity; moderate pH forms a continuous 3D network	DPD + all-atom MD; COMPASS force field; Flory-Huggins parameter mapping	[75]
Structure-regulating external field	Enhance mechanical strength; improve toughness; regulate swelling	Formation of a semi-interpenetrating network; enhanced chain entanglement, hydrogen bonding, and van der Waals interactions	All-atom MD simulation; GAFF AMBER force field	[76]
Magnetic field	Regulate swelling rate; increase ultimate strength (US)	Alternating magnetic field *B* = *B_0_sin(ωt)*; enhanced molecular vibration and collision; promoted polymer chain alignment	MD simulation; Lennard-Jones (LJ) and Coulomb force fields	[72]
Thermal field	Regulate molecular mobility; alter swelling rate; affect diffusion behavior	Elevated temperature enhances polymer chain vibration and reduces hydrogen-bond stability	MD simulation; Coulomb and Lennard-Jones (LJ) force fields	[77]
Thermal field + nanoparticle concentration field	Regulate water permeability; optimize drug release	Temperature increase enhances diffusion coefficient and water permeability; ZnO concentration mechanism	MD simulation; Lennard-Jones (LJ) force fields	[78]
Temperature field + mechanical stretching field	Regulate tensile strength and modulus; reveal temperature dependence	Temperature-driven mechanism; three-state transformation mechanism of water molecules	MD simulation; OPLS-AA force field	[79]
pH field	Regulate swelling rate; alter network stability	pH changes functional group protonation state; modifies interchain electrostatic repulsion/attraction; regulates polymer–water hydrogen bonding	MD simulation; GROMOS 54A7 force field	[80]
Thermal field + volume fraction field	Regulate swelling and diffusion performance; alter network stability	Elevated temperature increases hydrogen-bond breakage frequency and diffusion coefficient; modifies water–polymer interaction strength	MD simulation; CVFF, Lennard-Jones (LJ), and Coulomb force fields	[81]
Water content field + thermal field	Optimize mechanical performance; enhance interfacial bonding strength	Water content mechanism; temperature mechanism	MD simulation; COMPASS force field	[82]
Temperature field	Construct body-temperature-responsive hydrogel	Temperature effect mechanism; hydrogen bonding and van der Waals interactions	MD simulation; CHARMM36 force field combined with CGenFF	[83]
Temperature field	Control sustained drug release	Temperature mechanism; regulation of hydrogel network pore size	MD simulation; COMPASS force field	[84]

Regarding temperature and chemical environment as external regulatory factors, MD simulations indicate that the self−assembly process of peptide amphiphiles is synergistically governed by temperature and electrostatic repulsion strength. Together, these factors determine the evolutionary pathways and accessible conformational space of the system among spherical micelles, β−sheet−enriched nanofibers, and other intermediate or amorphous structures, thereby influencing the kinetic mechanisms of self−assembly rather than merely dictating a single final-state structure [85]. By introducing the low−molecular-weight cofactor cyanuric acid to “structurally reprogram” the noncanonical base pairing of oligo-adenine DNA, reversible formation of A−-motif duplexes, A−CA triplexes, or disassembled single−stranded structures can be achieved under different pH conditions, thereby constructing a supramolecular DNA hydrogel that combines strong acid resistance with physiological pH responsiveness. Density functional theory (DFT) and molecular dynamics (MD) simulations confirm at the molecular level that protonation/deprotonation−driven conformational transitions and their regulation of gel stability constitute the key mechanism underlying the reversible structural transformation of this system, enabling effective protection and controlled release of insulin within the gastrointestinal pH gradient environment [86].

In studies of low−temperature and antifreeze response mechanisms, a PVA/PHEAA double−network hydrogel model with both physical and chemical connectivity features can be constructed using the random walk reaction polymerization (RWRP) algorithm. MD simulations indicate that the antifreeze performance of this hydrogel primarily arises from strong interactions between the polymer network and water molecules, as well as the spatial confinement imposed by the double−network structure on water mobility. Under low−temperature conditions, hydrogen bonding between the polymer and water becomes more stable, the proportion of bound water increases significantly, and the diffusion and orientational relaxation of water molecules are markedly suppressed, leading to a substantial reduction in the accessible configurational space of water molecules. This confined water state, synergistically induced by network architecture and functional groups, is unfavorable for the formation of ice−like hydrogen-bond networks at both structural and dynamic levels, thereby competitively inhibiting ice nucleation and growth [87]. Zhou et al. developed an ionic hydrogel capable of ultrafast self−healing at −20 °C, with the core mechanism based on the synergistic regulation of dynamic chemical bonds and ion–hydration interactions. The introduction of Schiff base bonds and diselenide bonds provides reversible crosslinking sites, while the incorporation of LiCl suppresses freezing through strong hydration effects and maintains polymer chain mobility. MD simulations further confirm that Li^+^–water interactions significantly improve the dynamic behavior of water molecules under low−temperature conditions, thereby supporting rapid self-healing capability and stable ionic conductivity. Strain and pressure sensors constructed from this hydrogel exhibit excellent stability at low temperatures, demonstrating their potential applications in bioelectronics for extreme environments [88].

In studies of extreme environmental adaptability, the development of antifreeze hydrogels highlights the critical roles of water–ice phase equilibrium and confined water behavior. Existing research indicates that the antifreeze performance of hydrogels does not simply depend on reducing water content, but rather on the confined state of water molecules within the polymer network and their dynamic characteristics [89]. Current mainstream strategies mainly include the incorporation of antifreeze additives (such as inorganic salts, ionic liquids, organic solvents, or biomolecules), as well as restricting water migration by increasing crosslinking density, constructing microphase-separated structures, and optimizing hydrophilic-hydrophobic structural design. MD simulations have played an irreplaceable role in this field by revealing the molecular mechanisms through which confined water structures inhibit ice nucleation and recrystallization. However, maintaining the mechanical stability of hydrogels under low-temperature conditions remains a critical bottleneck limiting their practical applications. Future research should integrate molecular simulations with data−driven approaches such as machine learning to achieve synergistic optimization of network structure design and antifreeze performance regulation, thereby promoting the rational development of high-performance antifreeze hydrogels.

Beyond environmental adaptability, energy dissipation capacity is also a key performance metric for hydrogels in protective materials and flexible electronics. Huang et al. reported a high−damping hydrogel based on a microphase−separated double−network structure, whose design objective was not merely to enhance strength, but to achieve efficient energy dissipation through refined structural regulation. This system employs ultrasound to induce the formation of a PDMS microsphere network, combined with a physically crosslinked network formed by PVA through freeze–thaw cycles, resulting in a double−network structure with a bimodal particle size distribution. MD simulations indicate that enhanced interfacial friction between PVA chains and PDMS particles is the fundamental origin of the high loss factor (tan δ ≈ 0.51), while the bimodal size distribution further amplifies the interfacial frictional dissipation effect [90]. This study reveals, at the molecular scale, the intrinsic relationship between microphase structural characteristics and damping performance, providing new theoretical insights for the structural design of flexible protective materials and cushioning layers in electronic devices.

In summary, the response behavior of hydrogels under external fields and low−temperature conditions fundamentally arises from the cooperative rearrangement of water molecules, ions, and polymer chains under external stimuli, rather than from simple thermal effects or macroscopic structural changes. By elucidating, at the molecular level, how external fields modulate the system’s energy landscape and kinetic pathways, these studies provide a clear physical foundation and design principles for the development of electrically responsive, magnetically responsive, thermally responsive, and antifreeze hydrogels.

### 4.2. Multiscale Simulations Revealing Interfacial Behavior and Nonequilibrium Response Mechanisms of Hydrogels

**Figure 5:**
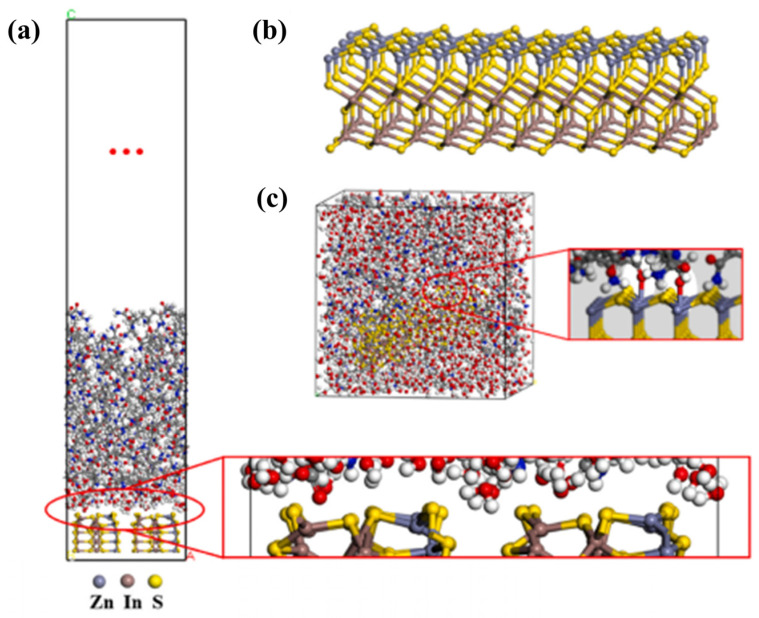
Multiscale simulation framework for hydrogel systems. (**a**–**c**) All-atom molecular dynamics simulation models of the ZIS–water–hydrogel interface and ZIS/PAM–water–hydrogel [36]. Reprinted with permission from Elsevier of Copyright 2025.

**Figure 6:**
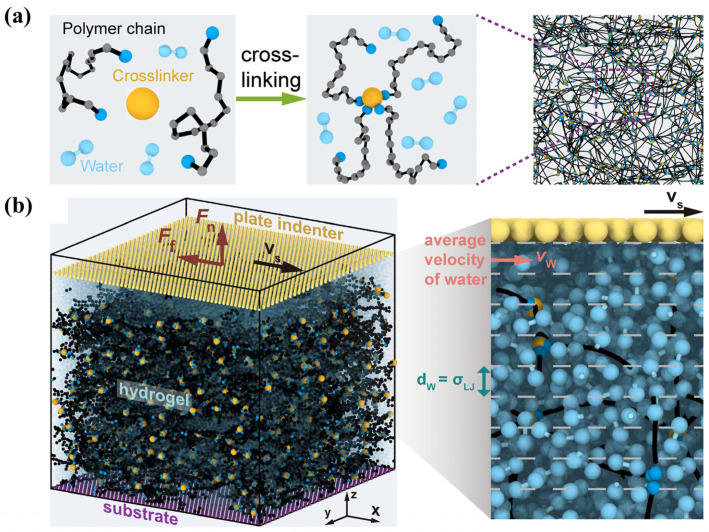
Schematic illustration of coarse-grained molecular dynamics simulations used to investigate the frictional properties of hydrogels [91]. (**a**) Illustration of the model hydrogel system preparation. The left panel depicts randomly dispersed precursor polymer chains (end monomers in dark blue, other monomers in gray), tetrafunctional crosslinkers (depicted as orange spheres), and water molecules (depicted as light blue dimers) in the system. The middle panel shows the crosslinking process between the end monomers and the crosslinker. The right panel displays the time-averaged structure of the model hydrogel after crosslinking. For clarity, water molecules are not shown in the right panel. (**b**) Illustration of the model system for evaluating hydrogel friction. The hydrogel was tethered to a substrate and then slid with a plate indenter. To determine the velocity of water molecules, the system was partitioned into layers along the z direction, each with a thickness of σ_LJ_. The velocity was then averaged over each layer, as illustrated in the magnified view in the right panel. Reprinted with permission from Wiley–VCH GmbH of Copyright 2025.

**Figure 7:**
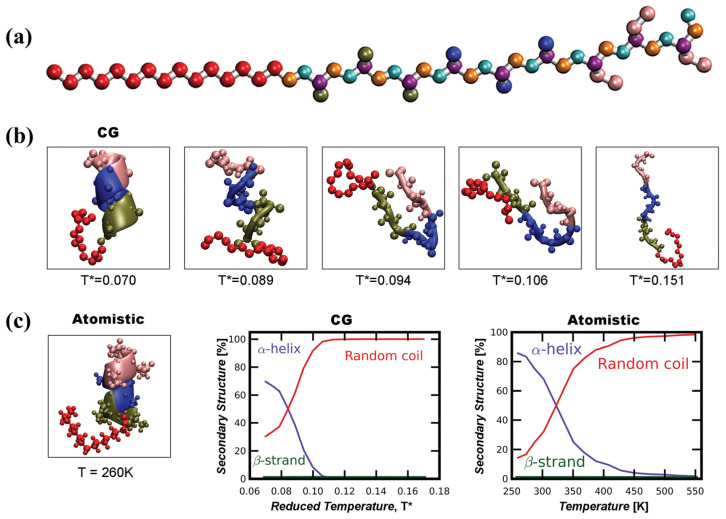
Comparison of results obtained from all-atom molecular dynamics and coarse-grained molecular dynamics simulations using peptide amphiphiles (PA) as an example [85]. (**a**) Geometry of the coarse–grained (CG) model, ePRIME, for the studied PA molecule, palmitoyl-Val_3_Ala_3_Glu_3_, representing united groups of atoms as spheres: alkyl group (red); valine sidechain (green), alanine sidechain (blue), glutamic acid sidechain (pink), and backbone atoms (NH, C_α_H, and CO as cyan, purple and orange, respectively). The united atoms are not shown at full size for ease of viewing. (**b**) Snapshots of equilibrium structures of the PA molecule at different reduced temperatures from ePRIME replica-exchange simulation. The color scheme is: hydrophobic alkyl tail (red), valine (green), alanine (blue), and glutamic acid (pink), consistent for all subsequent figures. (**c**) Equilibrium PA structure obtained from NAMD replica-exchange temperature simulation at *T* = 260 K. Plot of the percentage of secondary structure formation as a function of temperature obtained from replica-exchange simulations for 16 temperatures using coarse-grained ePRIME and atomistic NAMD. The temperature range for ePRIME is T* = 0.07–0.17 and for NAMD is *T* = 260–550 K. Data shown is taken from the average of the last 10% of equilibrium data and standard deviation is calculated to be ±20%. Reprinted with permission from WILEY–VCH Verlag GmbH & Co. KGaA, Weinheim of Copyright 2013.

With the increasing structural complexity and functional diversity of hydrogel systems, single−scale characterization or simulation methods are no longer sufficient to fully elucidate their underlying mechanisms. By combining MD simulations with electrochemical impedance spectroscopy experiments, researchers have established quantitative correlations among counterion migration behavior, interfacial ionic double-layer structural rearrangement, and macroscopic electroadhesion performance. This approach clarifies the synergistic regulatory mechanisms of ion diffusion rate, interfacial impedance, and double−layer capacitance in governing reversible electroadhesion behavior [64]. In addition, multiscale studies integrating experimental characterization, theoretical analysis, and coarse−grained simulations have shown that the time−dependent evolution of hydrogel friction performance in open−air environments arises from a nonequilibrium competitive process among water evaporation, internal water replenishment, and variations in the thickness of the hydration lubrication layer [91]. In drug delivery systems, molecular simulations of CSF/CF hydrogels formed by incorporating clemastine fumarate (CF) into hydrogel pores show that CF molecules can be uniformly distributed and stably embedded within the three−dimensional cavity structure formed by crosslinked fibers, thereby providing a structural basis for sustained drug release [92]. The sustained release of CF significantly protects the functions of endothelial cells and fibroblasts under high-glucose conditions, and promotes cell proliferation, migration, and angiogenesis by activating the MEK/ERK and PI3K/Akt signaling pathways, thereby markedly accelerating wound healing in diabetic rat models. A schematic illustration of the related multiscale simulation framework is shown in Figure 5, Figure 6 and Figure 7.

Using herbal-derived small molecules glycyrrhizic acid (GA) and puerarin (PUE) as low−molecular−weight gelators, a three−dimensional gel network can be orderly constructed through the synergistic driving forces of intermolecular hydrogen bonding, hydrophobic interactions, and π−π stacking. Small-angle X−ray scattering (SAXS) and molecular dynamics simulation results confirm that this system follows a co-assembly pathway. Its antibacterial activity does not originate from the intrinsic effect of a single molecule, but rather is determined by the specific nanostructures formed by GA−PUE aggregates, exhibiting selective inhibition against Staphylococcus aureus [93]. By employing the newly developed coarse−grained peptide/polymer model (ePRIME) in combination with discontinuous molecular dynamics (DMD), the complete kinetic pathways of peptide amphiphile self-assembly into different nanostructures can be captured starting from random initial configurations without presupposing any secondary structures. This strategy overcomes the time and length scale limitations of all−atom molecular dynamics and, through the construction of an electrostatic-temperature phase diagram, clarifies the kinetic mechanisms underlying morphology transitions under various external stimuli. It provides an operable theoretical framework for the application of intelligent self-assembled hydrogels in drug delivery, tissue engineering, and biomimetic material design [85]. In addition, all−atom molecular dynamics simulations indicate that different water states contribute significantly differently to the overall diffusion coefficient of water molecules in the system. Their weighted effects directly influence the transport efficiency of reactants such as protons and hydrogen, thereby exerting a critical constraint on photocatalytic performance [36].

In the field of hydrogel adhesion and adhesive materials, interfacial physicochemical mechanisms have been shown to be more critical than the intrinsic polymer structure [94,95,96]. Coarse−grained modeling and molecular dynamics simulations indicate that the influence of different polymer topologies (such as linear, star-shaped, brush−like, and dendritic structures) on hydrogel adhesion performance primarily depends on the total polymer density at the interface and the interfacial affinity between the polymer and the hydrogel substrate, with little significant correlation to structural parameters such as chain length, degree of branching, or topological geometry [97]. This phenomenon can be reasonably explained by scaling theory, in which the colloidal system is treated as an ideal ensemble of noninteracting linear chain segments. Further comparisons reveal that, compared with rigid nanoparticles, soft polymer particles are less likely to form repulsive inert layers at the interface, thereby exhibiting more robust and durable adhesion performance.

At the methodological level, MD simulations have become an important tool for characterizing the mechanical and thermal behaviors of pristine and nanocomposite hydrogels [98,99,100,101]. Relevant studies indicate that different crosslinking modes, spatial distributions of functional groups, and nanofiller–polymer interfacial interactions can significantly influence the molecular mechanisms underlying network mechanical responses. In elucidating these mechanisms, the appropriate selection of force field parameters and the construction of multiscale modeling strategies play crucial roles [102]. Moreover, in interfacial systems such as water/metal interfaces that exhibit strong electronic polarization effects, ab initio molecular dynamics possesses irreplaceable advantages in elucidating the coupling mechanisms between electronic structure and atomic motion [43]. However, its high computational cost significantly limits the accessible temporal and spatial scales. Therefore, it is necessary to integrate ab initio approaches with implicit solvent models or other approximate methods to effectively extend the simulation scale and applicability while maintaining physical reliability.

The Metropolis Monte Carlo simulation method based on explicit coarse−grained modeling, combined with the Weeks–Chandler–Andersen (WCA) repulsive potential for excluded−volume interactions, Coulomb electrostatic interactions, harmonic bond potentials, and Ewald summation, can capture two experimentally reported phenomena in microgel/nanogel composites: (1) a reduction in composite size even at relatively low nanoparticle (NP) content; and (2) a reversal of the electrophoretic mobility of the composites associated with charge inversion [103]. Yuji Higuchi et al. employed a coarse−grained molecular dynamics simulation approach to construct a double−network gel model consisting of a highly crosslinked first network and a lightly crosslinked second network, which was able to characterize the effects of structural parameters on tensile fracture behavior [104]. On this basis, the application of coarse−grained simulations has been further extended from conventional composite gels and double-network gels to topological gel systems with slidable crosslinking characteristics, enabling the characterization of how more complex network topologies regulate mechanical behavior. A coarse−grained (CG) model of topological gels was established based on the MARTINI force field to simulate their tensile behavior. The resulting CG model was able to accurately predict the elastic modulus of representative topological gels at different crosslinking densities [105].

As the research focus has shifted from the mechanical response of specific network structures to the accurate characterization of intrinsic material structures and interactions, the development of coarse-grained simulations has likewise progressed from phenomenological description toward force-field construction and parameterization optimization, with the aim of improving the physical realism and predictive capability of the models. A coarse−grained (CG) molecular dynamics force field for polyacrylamide (PAM), developed by fitting to the equation of state (EOS) derived from quantum mechanics (QM), was able to reproduce the EOS of PAM crystals, isolated PAM chains, and water–PAM systems, and successfully predict experimental properties such as density, specific heat capacity, thermal conductivity, and melting point [106]. A MARTINI coarse−grained (CG) force field, developed by combining all−atom molecular dynamics (AAMD) with the iterative Boltzmann inversion (IBI) method, enabled the construction of a mechanically high−fidelity polyacrylamide (PAAm) hydrogel model. This approach overcame the size limitations of traditional AAMD simulations and successfully revealed how polymer conformations drive the transition in hydrogel elasticity from the collapsed state to the swollen state [107]. Daniel G. Angelescu et al. proposed a coarse-grained parameterization model for phytic acid compatible with the Martini 2.3P force field, providing an effective tool for predicting the potential crosslinking modes and supramolecular structures of ionically crosslinked chitosan hydrogels. By coarse−graining both phytic acid and chitosan segments and optimizing the cross-interaction parameters between them, the study successfully reproduced the atomistic features of phytic acid-mediated crosslinking. The simulation results showed that the binding modes of phytic acid–chitosan complexes could reasonably explain the structural characteristics of crosslinked chitosan in semi−dilute solutions, and that the network topology and average pore size exhibited clear regulatory trends with changes in phytic acid concentration [108].

Furthermore, on the basis of constructing high-fidelity coarse−grained force fields, researchers have begun to introduce data-driven approaches and machine learning techniques into the coarse-grained modeling process to enable the efficient identification and rapid prediction of force-field parameters in complex crosslinked network systems. A multiscale strategy combining AAMD, CGMD, and machine learning, together with data-driven prediction of coarse−grained force-field (CGFF) parameters, can significantly improve the simulation efficiency of polymer complex crosslinked networks. Taking a typical chemically double−network hydrogel as an example, an artificial neural network was employed and trained using CGMD tensile stress–strain data generated with different CGFF parameters. The results showed that the CGMD simulations performed with the predicted CGFF parameters were in good agreement with the all-atom molecular dynamics (AAMD) simulation results, while the computational speed was improved by nearly 50−fold [109].

## 5. Conclusions

In summary, the structural formation, interfacial behavior, and service performance of hydrogels and nanocomposite hydrogels are essentially governed by the synergistic regulation of water, polymer chain segments, ions/functional groups, and multiscale interactions. With the rapid development of molecular dynamics simulations, coarse−grained methods, and multiscale coupled computations, researchers are now able to understand more systematically, at the atomic and molecular levels, the bulk network construction mechanisms of hydrogel systems, the interfacial charge–hydration synergistic effects, and the dynamic response behaviors under external fields and nonequilibrium conditions.

Regarding the bulk network and mechanical foundation, water molecules do not merely act as a solvent; rather, they are deeply involved in the hydrogen−bonding network, ionic associations, and chain rearrangement processes within hydrogels. Their molecular state and dynamic behavior directly affect network compactness, chain mobility, energy dissipation pathways, and macroscopic mechanical properties. Based on studies of zwitterionic hydrogel interfacial regulation and biofunctionality, zwitterionic motifs can form a dense and stable interfacial hydration layer through strong ion–dipole interactions, thereby endowing hydrogels with excellent interfacial bioinertness. Meanwhile, the network crosslinking density, incorporation of nanofillers, and spatial presentation of biomacromolecules further influence their functional performance in cell adhesion, migration, inflammation regulation, and the construction of tissue microenvironments. Regarding the dynamic response mechanisms under external fields and non−equilibrium conditions, multiscale simulations have shown that the interfacial behavior of hydrogels and their dynamic response mechanisms under non−equilibrium conditions are primarily governed by interfacial physicochemical factors. Among these, interfacial polymer density, interfacial affinity, and interfacial interactions are often more critical than the intrinsic topological structure of the polymers. Molecular dynamics and coarse−grained simulations have further revealed the decisive influence of crosslinking modes, functional group distribution, and nanoparticle–polymer interactions on the mechanical and thermal responses of the network. For complex interfacial systems with strong polarization effects, it is also necessary to combine ab initio molecular dynamics with multiscale approximation strategies in order to balance the accuracy of mechanistic analysis with the scalability of the simulation scale.

In the future, the development of multiscale simulation strategies integrating atomistic simulations, coarse−grained models, and continuum theories may help bridge the gaps in time and length scales. Deep coupling with in situ characterization techniques, as well as mechanical or electrical experiments, can enable closed-loop calibration between model parameters and predicted results. In addition, the incorporation of data-driven and machine learning methods is expected to accelerate the exploration of structure–property relationships, thereby enabling the efficient design and precise regulation of complex hydrogel systems.

## Figures and Tables

**Table 1:** Dynamic characteristics of water molecules in hydrogel systems and their regulatory factors.

Regulatory Factor	Key Function	Mechanism	Dynamic Impact	Reference
Gel microstructure and water migration	Water transport performance in hydrogels	Modification of microporous structure reduces diffusion resistance	Enhanced water absorption and desorption kinetics	[45]
Water–polymer hydrogen bonding and water state distribution	Characterization of bound/intermediate/free water states	Hydrogen bonding between water and polymer affects water molecule confinement	Bound water influences diffusion coefficient and dynamics	[46]
Crosslinking degree and ratio of free/bound water	Control of water states (freezable and non-freezable water)	DSC and NMR characterization of different water states	Changes in T_2_ relaxation and diffusion coefficient with crosslinking degree	[47]
Molecular structure of water and gel network	Structural and dynamic probing by IR/NMR	Molecular interactions between water and network	Affects water relaxation parameters and dynamics	[48]
Ionic strength and water-binding characteristics	Ions influence water binding and swelling	Salt ions alter the proportion of non-freezable water	Affects water diffusion and gel volume variation	[49]
Interfacial effects and water molecular structure	Orientation of interfacial water and electric field effects	Interfacial interactions induce water structure rearrangement	Electric potential leads to different interfacial water organization	[50]
Multiscale MD simulations revealing microscopic diffusion mechanisms	Combined MD and experimental analysis of dynamics	Simulation of hydrogen-bond networks between water and polymer	Reveals diffusion behavior of different water states	[51]
Swelling-induced interfacial stretching effects	Interfacial deformation affects water retention properties	Mechanical–water coupling induces interfacial instability	Swelling rate correlates with water transport	[52]

## Data Availability

No new data were created or analyzed in this study.
